# The largest known cowrie and the iterative evolution of giant cypraeid gastropods

**DOI:** 10.1038/s41598-020-78940-9

**Published:** 2020-12-14

**Authors:** Stefano Dominici, Mariagabriella Fornasiero, Luca Giusberti

**Affiliations:** 1 Museo di Storia Naturale, Università degli Studi di Firenze, Florence, Italy; 2 Museo di Geologia e Paleontologia, Università degli Studi di Padova, Padua, Italy; 3 Dipartimento di Geoscienze, Università degli Studi di Padova, Padua, Italy

**Keywords:** Biodiversity, Biogeography, Palaeontology, Evolutionary theory, Tectonics, Biodiversity, Biogeography, Evolutionary ecology, Macroecology, Palaeoecology, Ecology, Climate sciences, Climate change

## Abstract

Based on the fossil record, we explore the macroevolutionary relationship between species richness and gigantism in cowries (Cypraeidae), the best-studied family of gastropods, with a global diversity distribution that parallels that of tropical corals, mangroves and seagrasses. We introduce *Vicetia bizzottoi* sp. nov. based on a Priabonian fossil found in northeastern Italy, the largest documented cowrie found so far and the youngest of a lineage of Eocene Gisortiinae species. The Gisortiinae stratigraphic record in western Europe indicates that species selection favoured large size and armouring of the shell. Palaeoecology and per-stage species richness suggest that gigantism occurred in peripheral habitats with respect to diversity hotspots, where smaller species were favoured. The Eocene–Oligocene boundary was marked by a turnover and the Chattian global warming favoured small-sized species of derived clades. Species selection leading to gigantism is further documented in Miocene lineages of *Zoila* and *Umbilia*, in the southern hemisphere, two extant genera distributed at the periphery of modern diversity hotspots, suggesting that the negative relationship between size and diversity is a recurring pattern in the evolutionary history of cowries. This palaeontological evidence is projected onto the existing hypotheses that explain analogous biogeographic patterns in various other taxa. Likewise, body size-species richness negative relationship was possibly driven in cowries by physiological, ecological and life history constraints.

## Introduction

Covariance between community species richness and body size of resident species have been studied in a variety of marine invertebrates, suggesting different relationships of these two important ecological traits. While diversity generally decreases with latitude and depth^1,2^, in many ectotherms body size increases, although not all^3–6^. Clues among the Cypraeidae (cowries), the best-studied gastropods^7^, suggest that where diversity is highest species tend to be smaller. The hotspot of cypraeid diversity, for example, centred in the Indo-West Pacific (IWP^1,8^), does not yield the largest species, living instead in the Carribean Sea (*Macrocypraea cervus*, up to 190 mm)^7^. Large and very large cowries occupy deep habitats in western and southern coasts of Australia, on the opposite side of a sharp diversity cline that peaks in the Philippines, where up to more two hundred species of smaller forms live amidst a variety of reef organisms^8^. These are neighbouring, more than phylogenetically related^8–10^, with clades like the small-sized Erosariinae and Erroneinae experiencing the highest diversity^11^. The negative relationship among these ecological traits suggests either that an inherited propensity to gigantism was shared by some clade, but not by those that radiated at the tropics, or that the trait is controlled by ecological factors that favour relict clades of peripheral habitats, at higher latitudes or deeper and cooler waters with respect to centres of diversity^2^. The discovery of a 335 mm-long fossil of the largest cowrie ever to be documented, belonging to a new species of extinct genus *Vicetia,* extends our knowledge of gigantism in cowries of subfamily Gisortiinae. Recent taxonomic revisions of the European fossil record allow to frame this finding within the wider context of cypraeid diversity, shedding light on the nature of macroevolutionary pathways that led to a striking example of gigantism. The aim of this paper is to describe the new species and present a synthesis of the Eocene-Miocene distribution of European cowries, focusing on species richness and maximum reported size of each species, to test the consistency of an hypothesis of negative relationship between gigantism and diversity in this particular molluscan clade. The fossil record is then confronted with modern macroecological theory to explore what physiological, ecological and life history constraints regulated the evolution of gigantism among cowries.

### Geology and age

**Figure 1:**
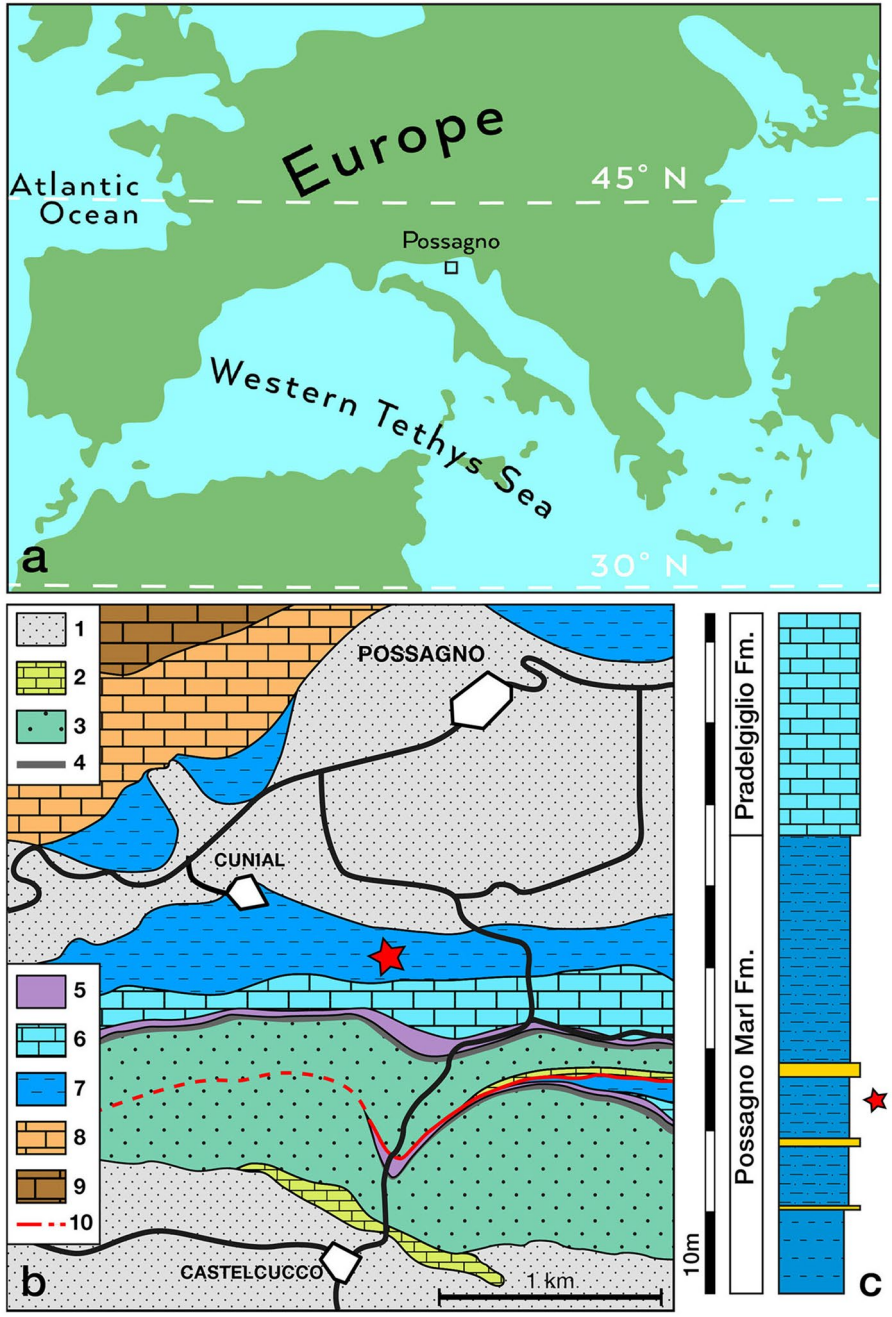
Location and geology of the Possagno area (northeastern Italy) and *Vicetia bizzottoi* fossil site. (**a**) Palaeogeography of western Europe with location of the study area. (**b**) Simplified geological map of the Possagno area with position of the *Vicetia bizzottoi* sp. nov. fossil site (red star). 1: Quaternary deposits; 2: Castelcucco calcarenite (Miocene); 3: glauconitic calcarenite (Miocene); 4: conglomerates (upper Oligocene-lower Miocene); 5: upper Possagno Marl Formation (upper Eocene); 6: Pradelgiglio Formation (upper Eocene); 7: Possagno Marl Formation (upper Eocene); 8: Scaglia Cinerea (middle-upper Eocene); 9: Scaglia Rossa (Upper Cretaceous-lower Eocene); 10: fault^75^. (c) Simplified stratigraphic log with position of the *Vicetia bizzottoi* sp. nov. bed (red star). Symbols for the Possagno Marl Fm. indicate marls and silty marls, in blue, and fine-grained sandstones, in orange. Maps created with Adobe Illustrator CC 2017 (21.0; https://helpx.adobe.com/it/illustrator/release-note/illustrator-cc-2017-21-0-release-notes.html).

The large specimen of *Vicetia bizzottoi* sp. nov. was found in the upper part of the Possagno Marl Formation near the Cunial quarry, Possagno (Treviso province, Veneto region of NE Italy). Gently dipping towards SSE, this unit is part of the monocline dividing the Mesozoic succession of the Monte Grappa massif, in the southeastern Alps, from the Venetian Plain. The sedimentary Priabonian succession cropping out at Possagno, about 45 km to the NW of Priabona, is the thickest expression of this time interval in the region and thus formerly proposed as parastratotype section of the Priabonian stage (upper Eocene^12^). During this interval Veneto formed part of a vast embayment in the western sector of the Tethys Sea, delimited to N and E by the European continent and to W and S by the early expression of the Apennine chain (Fig. 1a). The fossil was collected in grey mudstone, part of a coarsening-upward alternation of shelly mudstones with thinner sandstone intervals, about 30 m below the base of the overlying Pradelgiglio Limestone Formation^13^ (Fig. 1b,c). A skull of the sirenian *Prototherium intermedium* (specimen B^14^) was found in the same interval, together with bivalves *Pycnodonte gigantica*, *Chama granulosa* and *Crassatella* sp., gastropods *Ampullinopsis crassatina* and *Clavilithes yapeti* and solitary corals *Pattalophyllia subinflata*, *P. roseni*, *Placosmiliopsis fimbriatus*, *Wellsotrochus polygonata* and *Montanarophyllia exarata*^15^. The microfaunal assemblage includes fragments of regular echinoids and nummulitid and large miliolid foraminifera, suggesting depth within the photic zone, while the coral and otolith assemblages indicate an offshore shelf palaeoenvironment^16,17^.

## Results

### Systematic palaeontology

Class Gastropoda Cuvier, 1797.

Order Littorinimorpha Golikov and Starobogatov, 1975.

Family Cypraeidae Rafinesque, 1815.

Subfamily Gisortiinae Schilder, 1927.

Genus *Vicetia* Fabiani, 1905.

*Type species* *Ovula hantkeni* Lefèvre T., 1878; Monte Postale (Bolca), Italy; lower Eocene, upper Ypresian.

*Referred species* The type species, *Vicetia bellardii* (Deshayes in Bellardi, 1852); Palarea (Blausasc), France; middle Eocene, Bartonian; *Vicetia gennevauxi* (Doncieux, 1908); Coustouge (Corbières), France; lower Eocene, Ypresian; *Vicetia jamesi* (Vredenburg, 1927); Sind, Pakistan; middle Eocene; *Vicetia bizzottoi* sp. nov. Possagno, Italy; upper Eocene, middle Priabonian.

*Original diagnosis* Subcypraeiform, bulky, smooth, completely involuted shell; two bulgy transversal dorsal ridges; wide and slightly convex ventral side; long aperture, cut at the two extremities; denticulations on outer lip; anterior columellar fold with some denticulations^18^.

*Remarks Vicetia *has been considered synonym^19^ or subgenus^20^ of *Gisortia*, Jousseaume, 1884, type species *Cypraea coombii* J. de C. Sowerby, 1850. The general shape (barrel-like vs oval), the number of transversal dorsal ridges (two or one) and the shape of the aperture (sinuous and narrow vs more straight and opening anteriorly), allow to clearly separate two clades that deserve the status of separate genera, as recognised by recent literature^21^.

*Vicetia bizzottoi* sp. nov.

*LSID* urn:lsid:zoobank.org:act: BD76B5C2-3B4B-4D5E-8EC7-A6A2779F65DE.

*Etymology* Patronymic. The new species is dedicated to Mr. Bruno Bizzotto, discoverer and preparator of the type specimen.

**Figure 2:**
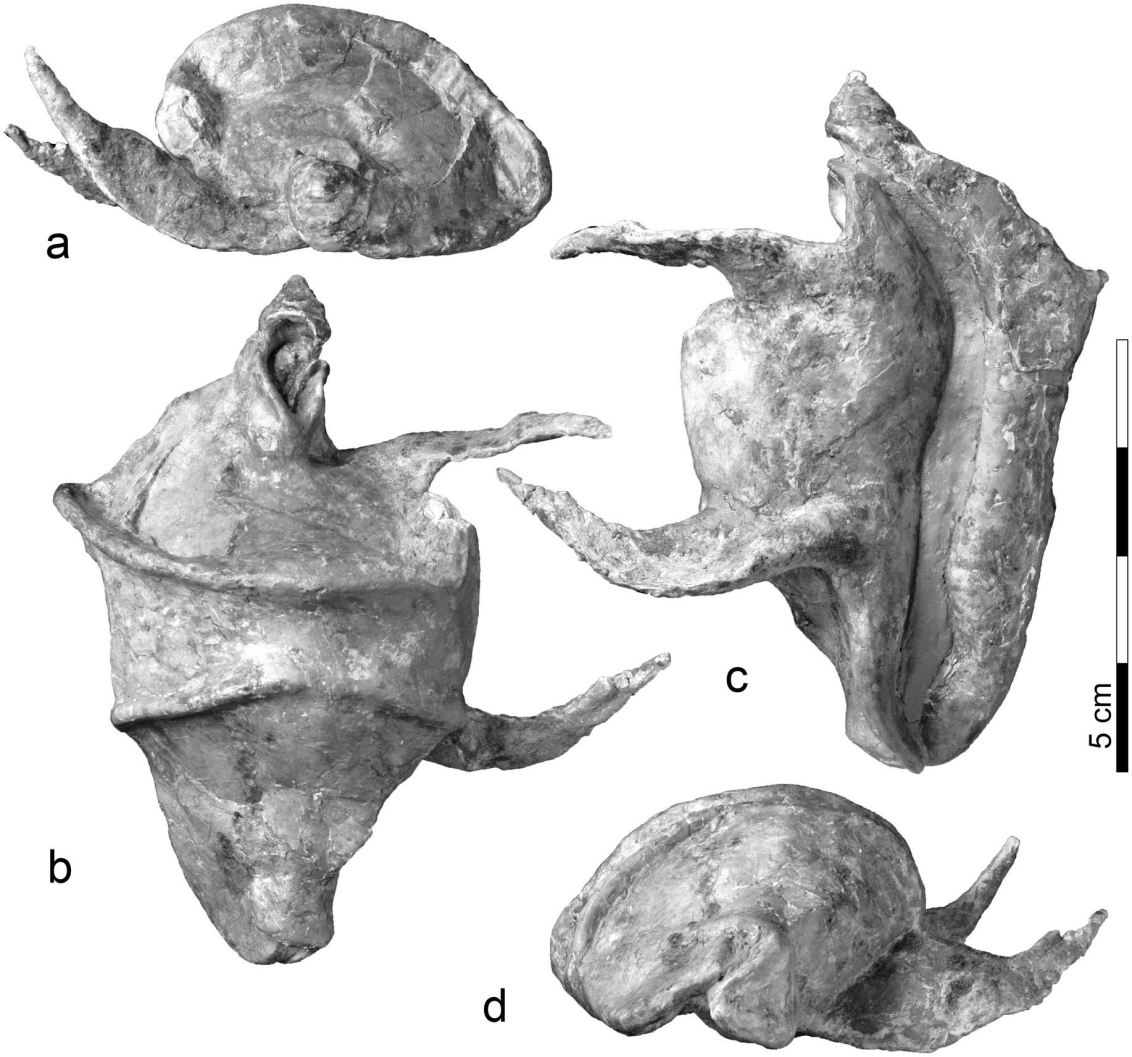
Shell of *Vicetia bizzottoi* sp. nov. (Holotype MGP-PD 32314). (**a**) Posterior view, (**b**) ventral view, (**c**) dorsal view, (**d**) anterior view.

*Holotype* MGP-PD 32314 (holotype by monotypy: Fig. 2).

*Material* The holotype MGP-PD 32314, housed in the Museo di Geologia e Paleontologia of the Padova University, Italy.

#### Locality and horizon

Only known from the vicinity of Cunial quarry fossil site, in Possagno, Treviso, in northeastern Italy, Possagno Marl Formation (middle-upper Priabonian, upper Eocene^22,23^).

#### Diagnosis

Two acute dorsal ridges that converge dorsally in the midline; two very long and pointed protuberances originating from the ventral side and extending to the left, curved towards the dorsal side; a twisted and adapically-protruding exhaling canal.

#### Differential diagnosis

The two acute dorsal ridges and the narrow and long aperture are consistent with the diagnosis of *Vicetia* and the referred specimen is here regarded as pertaining to a new species.

The type species of *Vicetia*, *V. hantkeni*, is well-known in the lower and middle Eocene of western Europe (Ypresian of Veneto and Friuli, Italy; Lutetian of France), also under the junior synonyms *Vicetia o’gormani* Cossmann, 1923^24^ and *Vicetia douvillei* Cossmann and Pissarro, 1911^21^. It differs for its much smaller size and the blunt dorsal ridges, more distant from one another than in *Vicetia bizzottoi* sp. nov. It lacks a protruding exhaling canal and has only one blunt and much shorter lateral protuberance.

*Vicetia gennevauxi* (Doncieux, 1908) is known only from the lower Eocene of the French Pyrenees (Ypresian of the Corbières, France). This is the smallest and less ornamented of all species of the group, assigned to the same genus for its two faint dorsal ridges.

The closest species is the middle Eocene *Vicetia bellardii* (Deshayes in Bellardi, 1952), found in the Lutetian of the Paris Basin and Bartonian of North (Val d’Oise) and South France (Alpes-Maritimes) and in the southern Pyrenees, in Spain, under the junior synonyms *Ovula gigantea hoernesi* Lefèvre, 1878, and *Gisortia vicetiana* (Farrés and Stadt 2009)^21^. Adult specimens are from large to very large (up to 280 mm), not attaining the size of *Vicetia bizzottoi* sp. nov. The two dorsal ridges are blunt and equidistant. Lateral protuberances are two and sharp, but very short and not pointed. The aperture is slightly wider and gently curved, not sinuous. Bartonian specimens from the Spanish Pyrenees have a long, but untwisted exhaling canal (or tubular anterior channel^24^).

#### Description

Gigantic cowrie, twice the average size of species of the same subfamily (based on length: Supplementary Table SI2); the posterior size of the shell is larger than the anterior; the aperture is narrow, sinuous and very long, with two convexities facing to the right; the exhaling canal is twisted and protruded, about one fifth of the whole shell length, slightly pointing to the left; the dorsal side is crossed by two acute ridges, coming closer towards the midline and distancing both to the left and the right side; on the right side of the shell the posterior ridge is more pronounced than the anterior, merging with the outer lip at about one third of its length; on the left side the two ridges are less pronounced and rapidly disappear ventrally. Two very long and pointed protuberances protrude from the ventral side of the shell; the anterior one merges with the inner lip at about two fifths of its length; the posterior protuberance disappears ventrally and anteriorly, but connects posteriorly with the anterior flank of the exhaling canal; the two protuberances are about as long as the body of the width of the shell at the point of junction; they arch dorsally and end with sharp points. Apart from the dorsally sharp ridges and protuberances, the surface of the shell is smooth.

## Discussion

### Stratigraphic distribution

**Figure 3:**
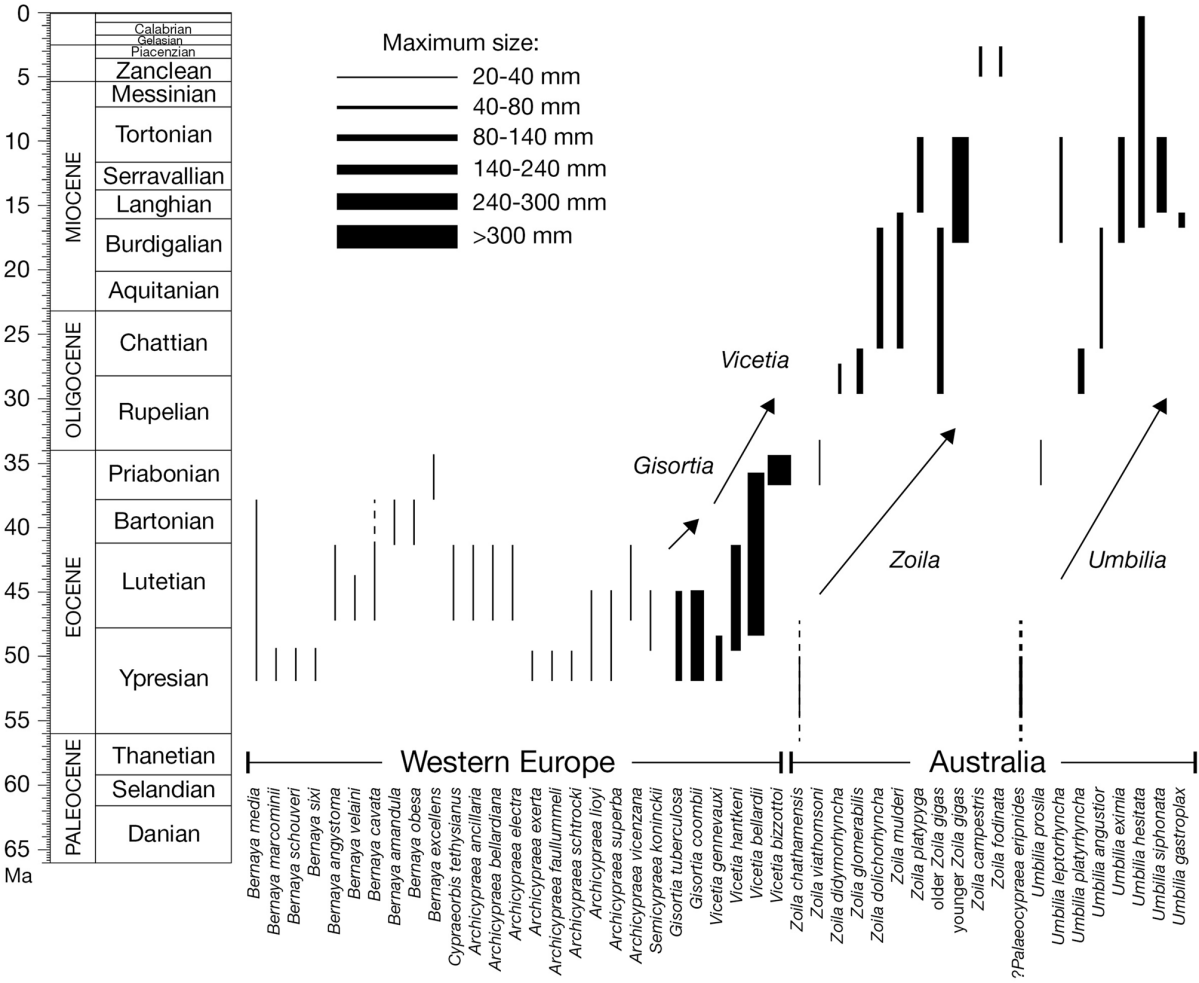
Stratigraphic range of Gisortiinae cypraeids in western Europe and genera *Zoila* and *Umbilia* in Australia. Maximum known shell size shown by line thickness. Arrows indicate trends in size increase recognised in successive species of four distinct genera.

**Figure 4:**
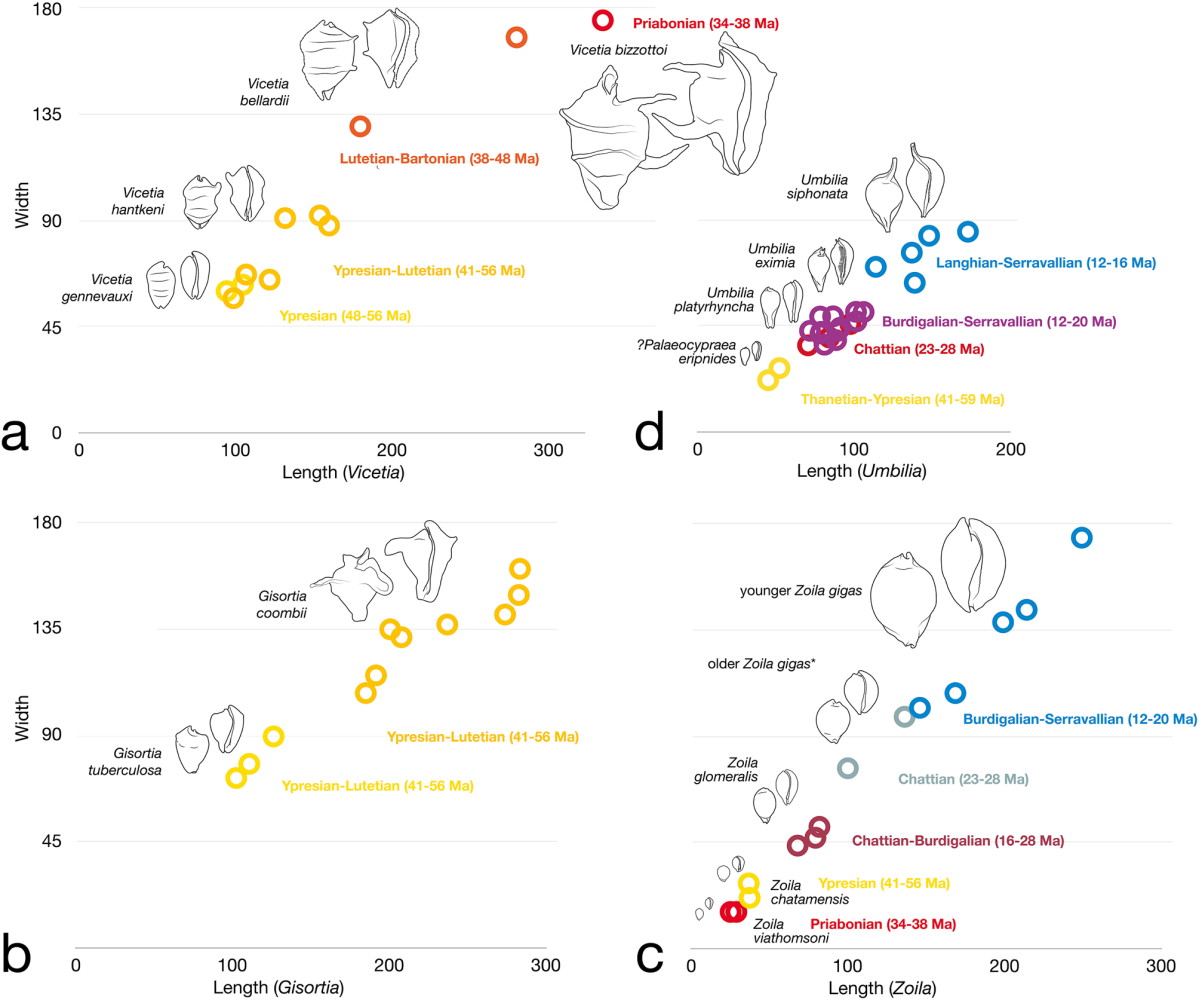
Lineages of cowries from the Eocene of western Europe (**a**,**b**) and the Cenozoic of Australia (**c**,**d**). (**a**) Four successive species of *Vicetia* show increase in size and armouring. (**b**) Two contemporaneous species of *Gisortia* have different size range and armouring. (**c**) Four species of the lineage that leads to middle Miocene *Zoila gigas*. *Chattian specimens, of smaller size and shorter canals, were previously attributed to different species (Supplementary Information SI1). (**d**) Species chosen to show net size increase the connects the small *Palaeocypraea*? *eripnides* to the middle Miocene *Umbilia siphonata*.

The fossil record of *Vicetia* in western Europe (England, France, Spain and Italy) has been recently reviewed^19,21,24^, resulting in the recognition of three species of late Ypresian-Bartonian age (early-middle Eocene), other names being placed in synonymy. A fourth species described here positively extends the record of the genus to the Priabonian (upper Eocene), so far known only from moulds of doubtful attribution (specimens from Lonigo and Brendola, two Priabonian localities not far from Possagno^22^). The stratigraphic range of the first three species partly overlaps (Fig. 3), but a distinct stratigraphic trend is detected (Fig. 4a), from the Ypresian relatively small-sized *Vicetia gennevauxi*, with no pronounced ornaments, to the Ypresian-Lutetian medium-sized *Vicetia hantkeni*, with blunt ornamentation and one lateral projection, to the middle Eocene large to gigantic *Vicetia bellardii* with blunt ornamentation and two projections, to the gigantic upper Eocene *Vicetia bizzottoi* sp. nov., with sharp and long ornaments. A parallel trend is seen in two species of the other gigantic genus *Gisortia* of western Europe. *Gisortia tuberculata* is mainly found in upper Ypresian beds of France and only rarely in lower Lutetian glauconitic beds of southern England^25,26^, whereas *Gisortia coombii*, present in the same beds and same localities, becomes distinctly more abundant and widespread in the Lutetian^25^. The two species have different size and ornamentation^25,26^, showing a tendency parallel and partly contemporaneous to species of *Vicetia* (Fig. 4b). Other genera of subfamily Gisortiinae, widespread in several basins of northwestern Atlantic and western Tethys^27^, are more diversified (*Bernaya*, *Archicypraea*), smaller than *Vicetia* and *Gisortia*, showing no increase in size in time (Fig. 3).

A second group of very large and gigantic fossil cowries is found in the Neogene of the southern hemisphere belonging to *Zoila* and *Umbilia*^28,29^. The stratigraphic distribution of the several species of these two genera shows net increase in size. From small early Eocene ancestors, successive *Zoila* species become larger during the Oligocene and early Miocene, until reaching the maximum size in the middle Miocene with *Zoila gigas* (Fig. 4c). A parallel trend is seen in *Umbilia*, attaining very large size and long siphons with *Umbilia siphonata*, passing through species of intermediate size (Fig. 4d).

### Habitats

Habitats and niches occupied by basal Gisortiinae *Gisortia* and *Vicetia* can be indirectly reconstructed from synecological and palaeonvironmental context and from functional morphology. *Gisortia coombii* and *G. tuberculata*, in association with large strombid *Hippocrenes amplus* and bivalves *Venericor plancosta* and *Venericardia acuticosta*^30^, are mainly known from the lower Lutetian Earnley Formation^25^, a glauconitic sandstone unit of the Braklesham Group (Sussex, England) deposited in a sediment-starved offshore setting^31–33^. A similar association is found in the lower Lutetian at Gisors, in the Paris Basin (France), where *Gisortia coombii* is associated with *Hippocrenes amplus*^34^ in the *Nummulites laevigatus* limestone, interpreted as a lower-shoreface deposit^35^. The same species is associated with *Sycostoma* *pyrenaica* and species of *Strepsidura* and *Volutilithes* in open-marine silty sandstones of the Esera valley (Aragon, Spain^36^), part of an oligotrophic ramp association^37^. A similar association is recognised in the Lutetian of Vic area (Catalonia, Spain), where *G. coombii* is associated with large gastropods (*Campanile*, *Velates*, strombids) and shark teeth^38^. *Vicetia hantkeni* occurs in upper Ypresian shelly *Alveolina* limestones at Monte Postale (Veneto, Italy), within a high-diversity association that includes abundant *Archicypraea lioyi* and herbivores (*Cerithium chaperi*, *Pseudobellardia gomphoceras*^39^). The Monte Postale *Alveolina* limestone is interpreted as part of a complex coralgal setting^40,41^. *Vicetia* cf. *hantkeni* occurs in association with abundant *Nummulites* and other large molluscs in the upper Ypresian of the Esera valley, in open shelf marls^37^. Very large specimens of *Vicetia bellardii* are recovered in Bartonian marls of the Vic area (Catalonia) in association with large gastropods (*Campanile*, *Velates*) and deeper subtidal forms such as *Discocyclina*, bryozoans and arbacid echinoderms^38^. At the antipodes with respect to western Europe, Australian fossil gisortiinae are mainly known from the Serravallian Muddy Creek Marl Member of the Port Campbell Limestone (Otway Basin, Victoria). This unit is associated with a well-diversified molluscan fauna^42^, including neogastropods *Serratifusus craspedotus* and *Hispidofusus senticosus*^43^ and abundant cetacean and shark remains^44^, overall indicating a starved offshore shelf setting. In conclusion, larger gisortine cowries have always preferred offshore-lower shoreface depths in sediment-starved shelves, in association with herbivore and carnivore gastropods, phototrophic larger foraminifera and azooxanthellate solitary corals. If we assign to past cowries the same diet of modern descendants, then *Vicetia* and *Gisortia*, like *Zoila*^45^, ate sponges preferably at 30–50 m depth^46^, competing for the same prey with other gastropods (e.g., Columbellidae^47^). If relying on algae, then they possibly competed with strombids and other very large gastropods. This description fits high levels of competition in a low productivity, high-diversity stable ecosystem. The role played by gisortine cowries was that of low-metabolism, vagile predators adapted to feed on an energy-poor sessile animal resource, eventually switching to even poorer vegetal substitutes. Much unlike the average predator, large size and ornamentation were adaptations for defence of a ‘gentle giant’^5^.

Modern cowries are successful predators of tropical seas where they prefer hard bottoms, extending their geographic distribution to temperate seas, where they gradually diminish in diversity and abundance^7^. Diversity trend parallels those of fishes and other predators, reflecting patterns of habitat-structuring organisms such as corals, mangroves and seagrasses^1^. In tropical habitats, small size of many cowrie species favours hiding during the day, moving and going out for prey only during the night. Favourite preys are sponges, hydroids, carrion, or algae^46–48^. All cypraeids undergo larval development, except *Zoila*, *Umbilia* and *Barycypraea* that develop without any pelagic phase. These three genera occupy marginal habitats with respect to IWP hotspot of diversity, the first two living subtidally up to 300 m-depth in temperate waters^45,46,49,50^, *Barycypraea* feeding intertidally during the day^45,51^. The three genera with a peripheral geographic distribution share basal positions in molecular phylogenies^9,10^, suggesting a phylogenetic relationship with basal gisortiinae like those found in Europe. Furthermore, living *Zoila* and *Umbilia* occupy offshore habitats similarly to *Gisortia* and *Vicetia* and direct development has been hypothesised for the two extinct European giants^42^.

### Macroevolution and biogeography

**Figure 5:**
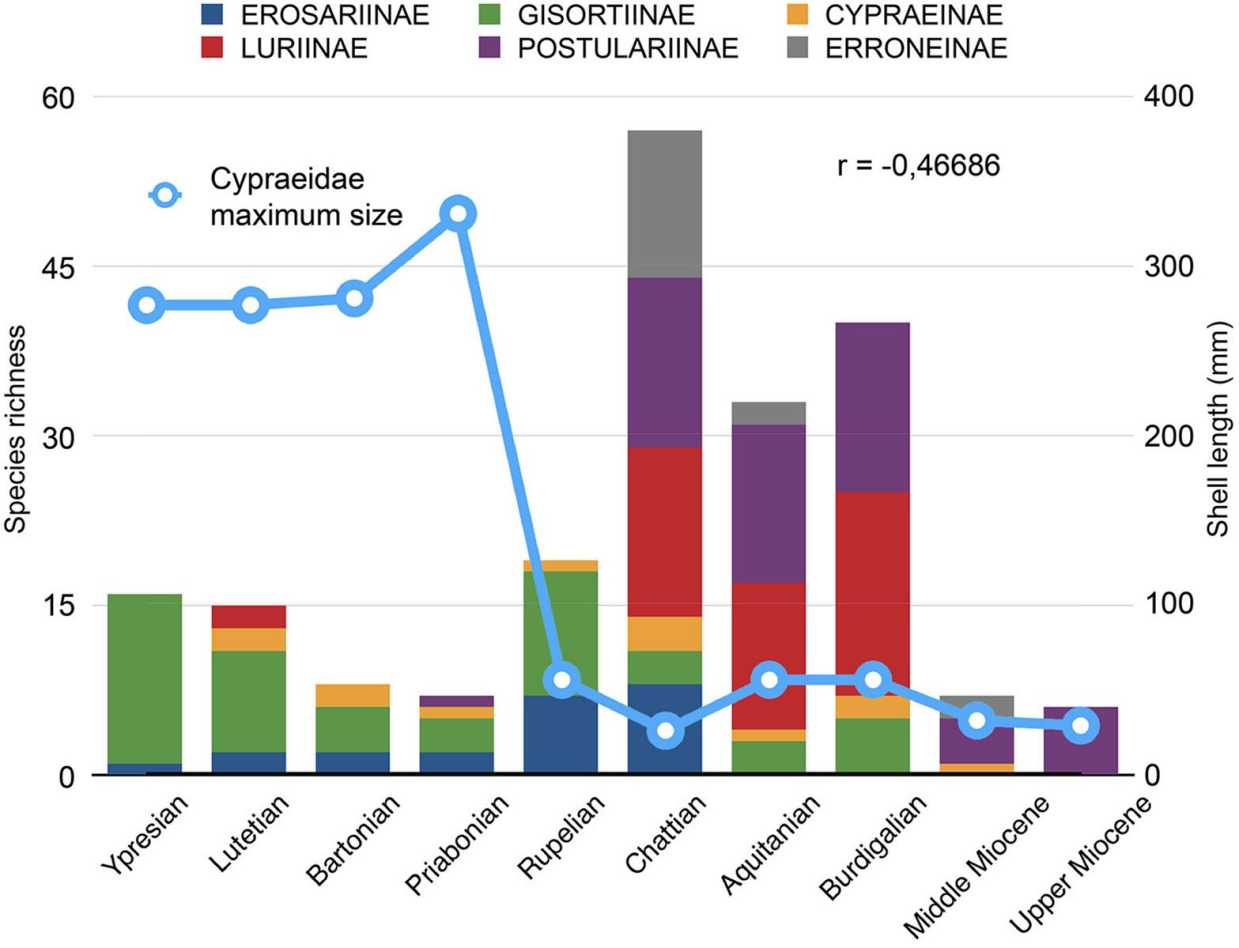
Species richness of western European cypraeid subfamilies during the Eocene-Miocene, compared to per-stage individual maximum size recorded in the family. The two values show a strong negative relationship (linear r = −0.46686), particularly for the Eocene-middle Miocene, before the closure with connections with IWP (linear r = −0.83099).

After the early Eocene climatic optimum, global temperature steadily decreased, until dropping at the end of the Priabonian^52^. Being unlikely that late-middle Eocene and late Eocene gigantic forms of France, Spain and Italy had migrated from the East, successive species of the same genus must have been connected by evolutionary relationships. The size increase seen in successive species of *Vicetia* (Fig. 4a) is thus an example of ‘differential success of species’^53^, or species selection^54^, reflecting the progressive and net macroevolutionary change in one lineage from large and un-ornamented cowries, to gigantic and sharply rostrate forms. We hypothesise that species of *Vicetia* were direct developers, like extant *Zoila*, *Umbilia* and *Barycypraea*, founder speciation promoting high speciation rate^8,55^. This line of descent is a candidate example of heterochrony through peramorphosis, or increased morphological development during ontogeny^56^, taking place by increase in growth rate of successive species and the progressively-earlier onset in the development of projections and ridges. The two species of *Gisortia* that co-occur with older species of *Vicetia* also suggest selection favouring larger species (Fig. 4b). The co-occurrence of up to four large or very large gisortiinae cowries during the Ypresian-early Lutetian, coupled to the high diversity of other genera of the same clade Gisortiinae, like *Bernaya* and *Archicyprea* (Fig. 3), is consistent with the hypothesis that the diversity hotspot at that time was centred in western Tethys^57–59^. Smaller species, associated with phototrophic corals and alveolinids (*Archycypraea lioyi* at Monte Postale^39,40^), decreased in diversity during the late-middle Eocene (Supplementary Fig. SI3). Offshore settings formed a refuge for larger remnants of the basal gisortines, as continental collision at 43 Ma and closure of northern seaways^60^ hindered biotic exchange with the IWP. Climatic cooling and the onset of the Antarctic ice sheet at the Eocene–Oligocene boundary^52^ coincides with a turnover at the level of subfamilies. In western Europe only two species of Gisortiinae are known, the clade still hosting the larger forms, while small and very small cowries sharply increase in species richness. The Oligocene diversity increase, a global phenomenon^25^, occurs in two steps. Basal forms (Erosariinae, genus *Proadusta*) are particularly diversified in the Rupelian (S_total_ = 24), derived subfamilies Luriinae, Pustulariinae and Erroneinae in the Chattian, as global temperature rises^52^ (S_total_ = 57: Fig. 5, Supplementary Figs. SI3–SI4). Evolutionary success of Oligocene cowries is measured by high species richness and by the highest percent of species with sizes not exceeding 20 mm (Fig. 5; Supplementary Fig. SI3). Cypraeids continued to be well-diversified during the warmer early Miocene, after which western Europe finally became disconnected^61^ from the IWP diversity hotspot^57^. As global climate progressively cooled^52^, middle and upper Miocene cowries decreased in richness (Supplementary Fig. SI3). Meanwhile at the other side of the globe, close to the new hotspot, a second rise to gigantism is observed in a clade reminiscing ancient Gisortiinae and including modern species of *Zoila* and *Barycypraea*. Both genera are direct developers that nest in molecular phylogenies^10^, one endemic to Australia^28,45^, the other surviving with two species in the western Indian Ocean^11^. Miocene species of *Barycypraea* show dorsal ornaments and a general shell-shape that suggests close phylogenetic relationships with Gisortiinae such as *Vicetia hantkeni*. They are particularly diversified in Java where they reach sizes of 60 mm. It is however the fossil record of *Zoila* that shows a pattern analogous to the *Vicetia* predecessor, consistent with a hypothesis of random speciation and channelling by differential success towards gigantic size (Fig. 4c). A fourth case concerns species of genus *Umbilia*, forming a lineage that leads not just to very large size, but also to the progressive elongation of the anterior canal (Fig. 4d) and the eventual widening of the peripheral region in the flanged cowrie, *Umbilia gastroplax*^29^. The dramatic global increase of productivity in coastal waters that occurred during the Cenozoic, greatly differentiates tropical habitats between the Eocene and the Miocene, favouring the ecological success of gigantic predators^62^. The similarities between Eocene and Miocene patterns leading to gigantism among cowries (Fig. 4) suggest phylogenetic constraints that control maximum body size and shell ornamentation, where climate change and plate tectonics preside over what traits are preferentially selected.

### Macroecology

Within-lineage body size increase over geologic time can be an effect of global cooling , as the macroevolutionary expression of the tendency for organisms to be larger in colder habitats^63,64^. The positive correlation between size and latitude, known as the temperature-size rule (TSR), is demonstrated for a large number of monophyletic clades of ectotherms^65,66^. This is not universal among benthic molluscs^6,67^, nor among all aquatic animals^68^, but recurring patterns in the cypraeid fossil record suggest that general causal TSR theories can be applied to the group, supported by experimental evidence on extant members of the family. By analogy with other modern lineages of ectotherms adapted to cold waters^4^, across-species body-size response of cypraeids was likely driven by selection acting on (1) physiological, (2) ecological, and (3) life history traits. (1) High ratio of oxygen availability at cold temperatures increases the window of body sizes into which lineages diversify and is suspected to be a general driving force of the TSR^66,69^. (2) Energetics of foraging behaviour—a largely-unexplored field in cowries—could also explain selection for larger body size^70^, with smaller cowries tending to graze sessile organisms like sponges, and larger ones accessing mobile prey. Getting bigger would be advantageous for catching escapers. Selection for starvation resistance, stronger in cold climates, also favours large size^66^. (3) The phenotypic plasticity in which ectotherms grow slower, but mature at a larger body size in colder environments, confirmed in cowries by laboratory rearing experiments^71^ and field surveys^72^, could be one driving force behind selection of larger forms during climatic cooling, at higher latitudes or in deeper waters. As an additional life history constraint, hatching at bigger would maximise fitness and be very important to getting a giant, shortening the period of growth. Bigger mothers would in their turn be advantageous for producing bigger eggs.

## Conclusions

The discovery of the gigantic cowrie *Vicetia bizzottoi* sp. nov. in Priabonian offshore deposits of western Tethys widens our understanding of factors that drove a lineage of Eocene Cypraeidae towards gigantism. Cowries have an excellent fossil record, a proxy useful to trace and interpret Cenozoic diversity hotspots. The well-known European record of subfamily Gisortiinae allows to reconstruct macroevolutionary patterns that characterise successive *Vicetia* species and contemporaneous species of sister genus *Gisortia*. Four Eocene species of *Vicetia* have a distribution consistent with an hypothesis of species selection leading to gigantism and increased ornamentation. Species of *Gisortia* experienced an analogous ecological success during the first part of the Eocene, before western Tethys became disconnected by plate tectonics from the Indian Ocean. Cypraeid diversity decreased both locally and globally, before a turnover at the Eocene–Oligocene transition replaced basal cowries with derived forms. Derived clades became highly diversified during the Oligocene, but mostly through small and very small species, both in basal and derived clades. New episodes of differential species selection towards gigantic size are documented by the Australian record of late Oligocene-middle Miocene genera *Zoila* and *Umbilia*. Analogies with Eocene gisortiines include offshore soft-bottom habitat and marginal position with respect to contemporaneous centres of diversity, where small cowries experienced very high species richness in communities from hard substrata. A conservative hypothesis is that the documented cases reflect convergent evolution in unrelated Palaeogene (*Gisortia* and *Vicetia*) and Neogene lineages (*Zoila* and *Umbilia*), favoured by direct development and high speciation rates. This iterative pattern characterised cypraeid giants notwithstanding the Eocene and Miocene experienced different regimes of productivity. The fossil record suggests that gigantism is confined within Eocene Gisortiinae of western Tethys and modern IWP relict and peripheral clades, possibly belonging to the same lineage. Palaeontological patterns conform to a general theory of temperature-size rule, where the rise of giants among cowries is driven by the interplay of factors that pertain to their physiology, ecology, and life history traits, shaped on a macroevolutionary scale by climate change and plate tectonics.

## Methods

The studied specimen was dug up and prepared using the usual palaeontological techniques. Species distributions and per-species maximum sizes were taken from updated palaeontological literature. Taxonomy follows the World Register of Marine Species^73^. Species richness was computed by artificially extending the stratigraphic range to the stage where each fossil was found and by including intervening stages in the case of discontinuous distributions. Correlation between per-stage species richness and maximum shell length was measured by linear r (Pearson’s coefficient) using the software PAST^74^.

### Nomenclatural acts

The electronic edition of this article conforms to the requirements of the amended International Code of Zoological Nomenclature, and hence the new names contained herein are available under that Code from the electronic edition of this article. This published work and the nomenclatural acts it contains have been registered in ZooBank, the online registration system for the ICZN. The ZooBank LSIDs (Life Science Identifiers) can be resolved and the associated information viewed through any standard web browser by appending the LSID to the prefix “http://zoobank.org/”. The LSID for this publication is: urn:lsid:zoobank.org:pub: BF3A9140-BDBB-4074-B3C7-39616E533574.

## Supplementary information

Supplementary Information.

## Data Availability

All data generated or analysed during this study are included in this published article and its supplementary information files.
