# Supplementary material for: The largest known cowrie and the iterative evolution of giant cypraeid gastropods
Source: Sci Rep. 2020 Dec 14;10:21893. doi: 10.1038/s41598-020-78940-9 (PMC7736312; doi:10.1038/s41598-020-78940-9)
Supplement: Supplementary file 1 — Supplementary Information. [file 41598_2020_78940_MOESM1_ESM.pdf]

Supplementary information

THE LARGEST KNOWN COWRIE AND THE ITERATIVE EVOLUTION OF GIANT CYPRAEID GASTROPODS

by STEFANO DOMINICI[1*], MARIAGABRIELLA FORNASIERO[2] *and* LUCA GIUSBERTI[3]

[1*]Museo di Storia Naturale, Università degli Studi di Firenze; e-mail: stefano.dominici@unifi.it

[2]Museo di Geologia e Paleontologia, Università degli Studi di Padova

[3]Dipartimento di Geoscienze, Università degli Studi di Padova

Supplementary Information SI1 We based "older *Zoila gigas*" in figs 3-4 on *Cypraea dorsata* Tate, 1890, collected in the Muddy Creek Marl and interpreted as a juvenile of *Zoila gigas* (McCoy, 1867) (specimen SAM T849[1], Fig. 13G-H; lower-middle Miocene[2]), and *Cypraea gabrieli* Chapman, 1912, also interpreted as a juvenile of *Z. gigas* (specimen P12366[1], Fig. 14A-B) from the Jan Juc Formation, collected at Bird Rock cliffs, Torquay (Australia, upper Oligocene[3]). These specimens differ from typical adults of *Zoila gigas*, ("younger *Zoila gigas*" in figs 3-4, based on specimens[1] in Figs 14D-E, 15) by the smaller size and shorter and less prominent anterior and posterior canals. Smaller specimens (L = 99-145 mm) are thus associated with older sediments (Oligocene-lower Miocene), whereas larger ones (up to 167-245 mm) are found in the Balcombian-Bairnsdalian[1,4] corresponding to the upper Burdigalian-Serravallian[5].

Supplementary Table SI2 Length (mm) of studied Gisortiinae specimens. Host institution and catalogue number indicated whenever possible (otherwise see references).

Species	Hosting institution	Catalogue number	Reference	Length (mm)
Vicetia hantkeni	Museo di Geologia e Paleontologia, Firenze	IGF 4499E	6	101
Vicetia hantkeni	Museum National d'Histoire Naturelle, Paris	MNHN.F.J05250	7	122
Vicetia hantkeni	Museum National d'Histoire Naturelle, Paris	MNHN.F.J03301	7	132
Vicetia hantkeni	Museo di Geologia e Paleontologia, Padova	MGP-PD 22083	8-10	99
Vicetia hantkeni	Museo di Geologia e Paleontologia, Padova	MGP-PD 32403	10-11	131
Vicetia hantkeni	Museo di Geologia e Paleontologia, Padova	MGP-PD 22082	11	154
Vicetia hantkeni	Museo di Geologia e Paleontologia, Padova	MGP-PD 1303R	Unpublished	189
Vicetia hantkeni	Museo di Geologia e Paleontologia, Padova	MGP-PD 1304R	Unpublished	93
Vicetia hantkeni	Unknown	[Coll. Cabassi]	8	160
Vicetia hantkeni	Museum National d'Histoire Naturelle, Paris	[Coll. Cossmann]	12-13	107
Vicetia gennevauxi	Unknown	[Lyon]	14-15	105
Vicetia gennevauxi	Museum National d'Histoire Naturelle, Paris	MNHN.F.R64165	14-15	95
Vicetia bellardii	Museum National d'Histoire Naturelle, Paris	MNHN.F.A53140	13	180
Vicetia bellardii	Museum National d'Histoire Naturelle, Paris	MNHN.F.A47970	13,16	280
Gisortia coombi	Museum National d'Histoire Naturelle, Paris	MNHN.F.R62966 [c]	15	273
Gisortia coombi	Museum National d'Histoire Naturelle, Paris	MNHN.F.R11876 [c]	15	264
Gisortia coombi	Museum National d'Histoire Naturelle, Paris	MNHN.F.B70330	15	200
Gisortia coombi	Museum National d'Histoire Naturelle, Paris	MNHN.F.R64617 [c]	15	193
Gisortia coombi	Museum National d'Histoire Naturelle, Paris	MNHN.F.R64942	15	228
Gisortia coombi	Museum National d'Histoire Naturelle, Paris	MNHN.F.R63200 [c]	15	178
Gisortia coombi	Museum National d'Histoire Naturelle, Paris	MNHN R64907 [c]	17	184
Gisortia coombi	Institut Royal des Sciences Naturelles de Belgique, Bruxelles	IRScNB C.I.6021	18	272
Gisortia tuberculosa	Natural History Museum, London	BMNH 47807	18-19	106
Gisortia tuberculosa	Museum National d'Histoire Naturelle, Paris	MNHN.F.R63076	18	98
Gisortia tuberculosa	Museum National d'Histoire Naturelle, Paris	MNHN.F.R64636	18	121
**Average length:**				163

Supplementary Figures

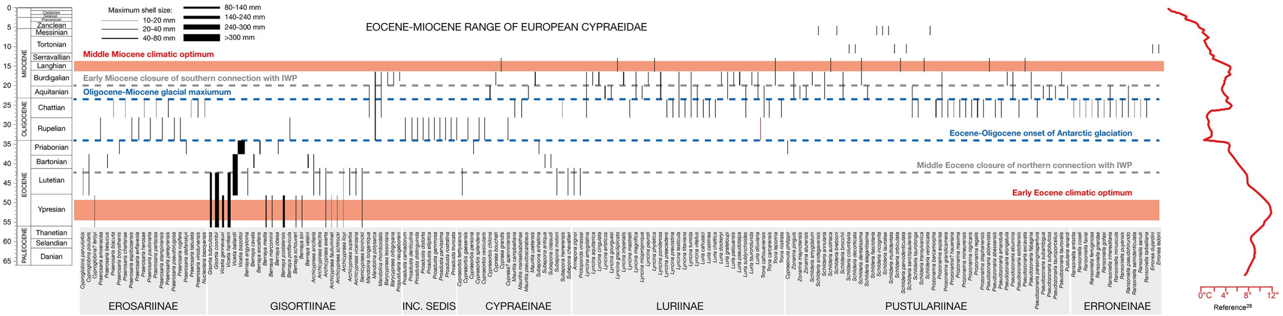

Supplementary Figure SI3. Eocene-Miocene distribution of western Europe species of Cypraeidae. Occurrences were extended to the whole stage and, in cases of gaps, to intervening stages. When data on size were not available (a minority of cases), line thickness corresponds to the 20-40 mm size range. Subfamilies follow recent taxonomies[21-23]: Erosariinae mainly includes species of *Praerosaria* and *Nucleolaria* (*Cyproglobina* included with doubt); Gisortiinae mainly includes species of *Gisortia*, *Vicetia*, *Bernaya*, *Archicypraea* and *Mandolina* (*Barycypraea* included with doubt); *Proadusta* is Incertae Sedis; Cypraeinae includes *Cypraeorbis* and *Mauritia* (*Subepona* could belong to the Erosariinae); Luriinae includes genera *Anepona*, *Protoponda*, *Lyncina*, *Luria* and *Trona*; Pustulariinae includes *Cypraeovula*, *Zonaria*, *Schilderia*, *Protozonaria*, *Pseudozonaria* and *Pustularia*; Erroneinae includes *Erronea* and, with doubt, *Ransoniella* (=*Plaziatia* Dolin & Lozouet, 2004). Data from France[17-18,20,24-25], Italy[7,13,26] and Ukraine[27]. Climatic events and temperatures from[28].

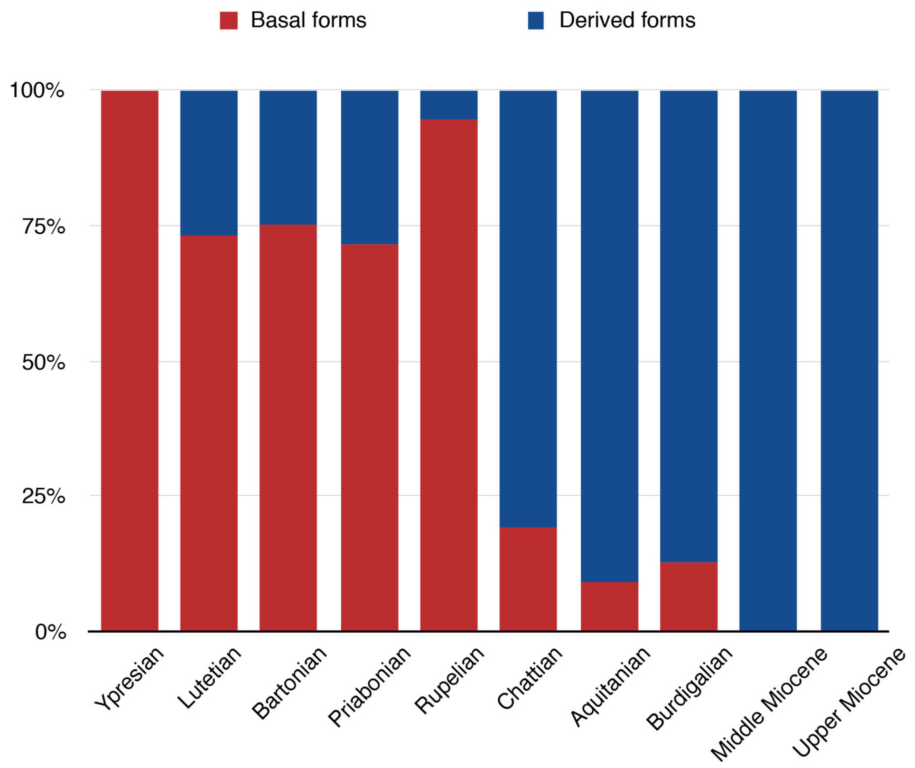

Supplementary Figure SI4. Western Europe per-stage cypraeid species richness expressed in percent of total, confronting basal Cypraeidae (subfamilies Erosariinae and Gisortiinae) with derived forms (Cypraeinae, Luriinae, Pustulariinae and Erroneinae). Data as in SI3.

References

1. Darragh, T.A. A revision of the Australian fossil species of *Zoila* (Gastropoda: Cypraeidae). *Memoirs of Museum Victoria* **68**, 1–28 (2011).
2. Fitzgerald E.M.G. A review of the Tertiary fossil Cetacea (Mammalia) localities in Australia. *Memoirs of Museum Victoria*, **61**, 183–206 (2004).
3. Li, Q., Davies, P.J. & McGowran, B. Foraminiferal sequence biostratigraphy of the Oligo-Miocene Janjukian strata from Torquay, southeastern Australia. *Australian Journal of Earth Sciences*, **46**, 261–273 (1999).
4. Pacaud, J.-M. & Loubry, P. *Zoila gigas* (McCoy, 1867) (Mollusca, Cypraeidae) le géant du Cénozoïque d'Australie. *Xenophora*, **138**, 34-36 (2012).
5. Allaby. M. *A Dictionary of Earth Sciences*. Oxford University Press, Oxford, 654 p. (2008).
6. Dominici, S. *Paleoecologia e stratigrafia sequenziale dell'Allogruppo di Castigaleu nell'area compresa tra Bacamorta e Val Esera (Eocene Inferiore, Pirenei centro-meridionali, Spagna)*. Unpublished doctoral thesis, Università di Parma, Italy, 1–94 (1994).
7. Dolin, L. and Pacaud, J.-M. Les Cypraeoidea et Velutinoidea (Mollusca, Caenogastropoda) du Lutétien inférieur du Vicentin et du Véronais (nord-est de l'Italie). *Revue de Paléobiologie* **28**, 277–314 (2009).
8. Fabiani, R. I molluschi eocenici del Monte Postale conservati nel Museo di Geologia della Università di Padova. *Atti dell'Accademia scientifica veneto-trentino-istriana* **2**: 145–158 (1905).
9. Cossmann, M. *Essais de Paléoconchologie comparée 7e livraison*, Ed. l'Auteur, Paris, 261 p, 14 pls. (1906).
10. Brigantini, T. Cypreidi, Naticidi e Olividi (Gastropodi) del Cenozoico nell'Italia nordorientale. *Memorie degli Instituti di Geologia e Mineralogia dell'Università di Padova*, **37**, 407-422 (1985).
11. Malaroda, R. Il Luteziano di Monte Postale (Lessini medî). *Memorie degli Istituti di Geologia e Mineralogia dell'Università di Padova*, **19**, 1-108 (1954).

12. Cossmann, M. & Pissarro, G. *Iconographie complète des coquilles fossiles de l'Eocène des environs de Paris*. Tome 2, Ed. Hermann, Paris, pls 26-45 (1911).
13. Pacaud, J.-M. Remarques taxonomiques et nomenclaturales sur les mollusques gastéropodes du Paléogène de France et description d'espèces nouvelles. Partie 2. Caenogastropoda (partim). *Cossmanniana* **21**, 101–153 (2019).
14. Doncieux L. Catalogue descriptif des fossiles nummulitiques de l'Aude et de l'Hérault. Deuxième partie (fascicule 1): description paléontologique du Nummulitique des Corbières septentrionales. *Annales de l'Université de Lyon*, **22**, 1-250 (1908).
15. Pacaud, J.-M. *Gisortia (s.str.) coombii* (J. de C. Sowerby in Dixon, 1850) une espèce emblématique de l'Eocène du bassin de Paris. *Fossiles*, 82-85 (2012).
16. Farrés, F. & Staid-Staadt, J.L. Cipréidos gigantes del Eoceno catalán. *Batalleria*, **14**, 57-66 (2009).
17. Pacaud, J.-M. Sur les spécimens types et figurés des taxons relatifs à *Gisortia* (s.str.) *coombii* (Sowerby in Dixon, 1850) (Gastropoda: Cypraeoidea). *Cossmanniana* **12**, 1–45 (2008a).
18. Pacaud, J.-M. L'original de *Gisortia gigantea pterophora* Schilder, 1927 (Mollusca, Gastropoda, Cypraeoidea) retrouvé. *Cossmanniana* **12**, 47–53 (2008b).
19. Pacaud, J.-M. & Canevet, J.-M. Considérations sur l'espèce *Ovula tuberculosa* Duclos, 1825, du Cuisien (Yprésien, Éocène inférieur du Bassin de Paris. *Saga Information* **372**, 61–66 (2019).
20. Dolin, L. & Aguerre, O. Les Cypraeidae et les Ovulidae (Mollusca: Caenogastropoda) du Cuisien (Yprésien moyen) du bassin de Paris (nord-ouest de la France). *Cossmanniana* **18**, 3–37 (2016).
21. Meyer, C.P. Molecular systematics of cowries (Gastropoda: Cypraeidae) and diversification patterns in the tropics. *Biological Journal of the Linnean Society* **79**, 401–459 (2003).
22. Meyer, C.P. Toward comprehensiveness: increased molecular sampling within Cypraeidae and its phylogenetic implications. *Malacologia* **46**, 127–156 (2004).
23. MolluscanBase, www.molluscabase.com (retrieved 23 July 2020).
24. Dolin L. & Lozouet, P. Nouvelles espèces de Gastéropodes (Mollusqua: Gastropoda) de l'Oligocène et du Miocène inférieur de l'Aquitaine (Sud-Ouest de la France). Partie 3. Cypraeidae et Ovulidae. *Cossmanniana* **4**, 1–164 (2004).
25. Pacaud, J.-M. *Bernaya amandula* nov. sp., une espèce nouvelle de Cypraeidae (Mollusca, Gastropoda) du Bartonien (Eocène Moyen) du bassin de Paris. *Fossiles* **34**, 52–57 (2018a).
26. Zamberlan, F. & Checchi, A. Il genere *Bernaya* Jousseaume, 1884 (Mollusca, Gastropoda, Cypraeoidea) dell'Eocene di "Cava Rossi" di Monte di Malo (Vicenza, Italia Nord-Orientale). *Studi e Ricerche Associazione Amici del Museo Civico "G. Zannato"* **21**, 17-27 (2014).
27. Pacaud, J.-M. Les Cypraeoidea (Mollusca, Caenogastropoda) du Priabonien (Éocène supérieur) de Dnipro (Oblast de Dnipropetrovsk, Ukraine). Partie 1: Cypraeidae. *Xenophora Taxonomy* **20**, 14–33 (2018b).
28. Zachos, J., Pagani, M., Sloan, L., Thomas, E., & Billups, K. Trends, rhythms, and aberrations in global climate 65 Ma to Present. *Science* **292**, 686–693 (2001).
